# Exploring Uncoupling Proteins and Antioxidant Mechanisms under Acute Cold Exposure in Brains of Fish

**DOI:** 10.1371/journal.pone.0018180

**Published:** 2011-03-25

**Authors:** Yung-Che Tseng, Ruo-Dong Chen, Magnus Lucassen, Maike M. Schmidt, Ralf Dringen, Doris Abele, Pung-Pung Hwang

**Affiliations:** 1 Institute of Cellular and Organismic Biology, Academia Sinica, Taipei City, Taiwan; 2 Leibniz-Institute of Marine Sciences, IFM-GEOMAR, Biological Oceanography, Kiel, Germany; 3 Institute of Zoology, National Taiwan University, Taipei City, Taiwan; 4 Alfred-Wegener Institute for Polar and Marine Research, Bremerhaven, Germany; 5 Center for Biomolecular Interactions Bremen, University of Bremen, Bremen, Germany; Texas A&M University, United States of America

## Abstract

Exposure to fluctuating temperatures accelerates the mitochondrial respiration and increases the formation of mitochondrial reactive oxygen species (ROS) in ectothermic vertebrates including fish. To date, little is known on potential oxidative damage and on protective antioxidative defense mechanisms in the brain of fish under cold shock. In this study, the concentration of cellular protein carbonyls in brain was significantly increased by 38% within 1 h after cold exposure (from 28°C to 18°C) of zebrafish (*Danio rerio*). In addition, the specific activity of superoxide dismutase (SOD) and the mRNA level of catalase (CAT) were increased after cold exposure by about 60% (6 h) and by 60%–90% (1 and 24 h), respectively, while the specific glutathione content as well as the ratio of glutathione disulfide to glutathione remained constant and at a very low level. In addition, cold exposure increased the protein level of hypoxia-inducible factor (HIF) by about 50% and the mRNA level of the glucose transporter *zglut3* in brain by 50%–100%. To test for an involvement of uncoupling proteins (UCPs) in the cold adaptation of zebrafish, five UCP members were annotated and identified (*zucp1-5*). With the exception of *zucp1*, the mRNA levels of the other four *zucps* were significantly increased after cold exposure. In addition, the mRNA levels of four of the fish homologs (*zppar*) of the peroxisome proliferator-activated receptor (PPAR) were increased after cold exposure. These data suggest that PPARs and UCPs are involved in the alterations observed in zebrafish brain after exposure to 18°C. The observed stimulation of the PPAR-UCP axis may help to prevent oxidative damage and to maintain metabolic balance and cellular homeostasis in the brains of ectothermic zebrafish upon cold exposure.

## Introduction

The vertebrate brain may be the organ most vulnerable to thermal fluctuations, since most of the physiological acclimation responses are initiated by the central nervous system (CNS) [1]. Cold shock causes severe pathologies in the mammalian brain [2], whereas mild cooling (to 32°C) can support survival and delay the deleterious effects of infarction [3]. Contrary, ectothermic vertebrates regularly experience brain cooling during day/night cycles of thermal fluctuations, or on seasonal scales, during winter in temperate regions. An early study on green sunfish (*Lepomis cyanellus*) demonstrates elevated activities of glycolytic enzymes, such as glucosephosphate isomerase, glyceraldehydephosphate dehydrogenase, and pyruvate kinase, in the brain during cold exposure [4]. The high metabolic rate of brain cells implies a high production of ROS [5]. In addition, homeoviscous adaptations, i.e. increased polyunsaturation of membrane phospholipids that maintain membrane fluidity in the brain during prolonged exposure to cold temperature [6], [7] enhance the susceptibility to oxidative stress [8]. Malek et al. [9] found a suite of antioxidant enzymes, including several superoxide dismutase (SOD) and glutathione peroxidase (GPx) isoforms and thioredoxin, but not catalase (CAT), up-regulated in zebrafish skeletal muscle, following temperature reduction from 28 to 18°C within 4 weeks and subsequent 6 months of maintenance at 18°C. In spite of the molecular antioxidant response, oxidative stress markers of lipid and protein oxidation and 8-oxodG-DNA damage were slightly higher in cold exposed zebrafish muscle. Thus, oxidative stress is indeed an issue during prolonged cold exposure in fish, which may also relate to the slow down of cellular repair mechanisms in the cold [10].

To avoid oxidative stress and keep cellular redox state in balance, aerobic cells utilize small molecular weight antioxidants and antioxidative enzymes. By activation of enzymes such as SOD, CAT, and peroxidases, cells respond to acute challenges. Low molecular weight antioxidants, such as ascorbate, glutathione (GSH), and phenolic compounds, contribute to the basal ROS scavenging antioxidant protection [11], often depending also on life history or feeding state.

Furthermore, “mild uncoupling” of the mitochondrial inner membrane controls the membrane potential and limits mitochondrial ROS production [12], [13], [14]. Oxidative phosphorylation is never fully coupled to ATP synthesis *in vivo*, conditional mainly on the existence of uncoupling proteins (UCPs) [15]. UCPs belong to a superfamily of mitochondrial anion-carriers (SLC25A) located in the mitochondrial inner membrane with a molecular mass of 31–34 kDa [16]. UCPs catalyze proton conductance and dissipate the mitochondrial gradient required for ATP production, which results in a reduction of mitochondrial membrane potential (ΔΨ_m_) and mitigates ROS formation, especially at respiratory complex I [17]. UCP activity is under close control of different effectors and inhibitors: it is strongly inhibited by purine nucleotides such as ATP and GDP and, in turn, activated by free-fatty acids and superoxide [18]. Moreover, the lipid peroxidation product 4-hydroxynoneal (HNE), also activates UCPs, and may serve as mediator of mild uncoupling, especially when mitochondrial membrane potential is high [12].

Five mammalian UCP homologues, UCP1-UCP5 are known. The original UCP, UCP1 (SLC25A7, thermogenin, 10% of membrane bound protein) is well known for its role in adaptive non-shivering thermogenesis (NST) in brown adipose tissue [19]. UCP2 is expressed in various tissues including brain, while UCP3 is expressed mainly in heart and skeletal muscles [20]. UCP4 (SLC25A27) and UCP5 (SLC25A14, also called BMCP1) are both expressed mainly in the nervous system, however, their functional characteristics are not well studied and their physiological roles are still unknown [15], [21]. The discovery of UCP homologues in ectotherms raises the question concerning their primary physiological role in thermoregulation [22], [23], [24], [25], [26]. On one hand, some piecemeal fish UCP paralogs have already been cloned [22], [24], [25]; on the other hand, in crocodile and several species of fish, mRNA levels or protein expressions of UCP homologues change in response to acute or seasonal temperature variations. Furthermore, the UCP genomic studies in fish and invertebrates raised an adaptive evolution scenario with respect to non-shivering thermogenesis (NST). Gene duplication and loss occurred in some lineages which was closely related to the UCPs' functional diversity between eutherians, the mammalian ancestors and ectotherms [27], [28].

PPARs (peroxisome proliferator-activated receptors) are ligand-activated transcription factors which belong to the nuclear receptor superfamily and regulate the expression of target genes involved in lipid and energy metabolism [29]. PPARs have been identified as pivotal actors in the control of UCPs gene transcription [30] in their respective tissues [31], [32]. In addition, PPARα also activates the expression of antioxidant enzymes, including SOD and GPx [33]. To date, three PPAR isoforms have been characterized in mammals: PPARα PPARβ/δ, and PPARγ, each isoform with a unique expression pattern relating to their distinct cellular functions [30].

Cold-induced oxidative stress has been suggested to play key role in brain damage. Neuronal UCPs are induced by oxidative stress products and by superoxide and seem to be crucial for reducing the mitochondrial ROS production [34]. The present paper is aimed at further investigating the neuroprotective effects of UCPs in fish brain, especially with respect to how UCPs are controlled under cold-induced oxidative stress in the fish CNS. The physiological role of PPARs in UCP gene expression and the mechanism of PPARs in the prevention of oxidative stress and neuroprotection have been reported in mammals [30], [31], [32], [33]. One of these studies suggests that UCPs may be involved in PPAR dependent gene transactivation through intrachromosomal looping next to their uncoupling function in the mitochondria [31]. Another important transcription factor involved in temperature control of gene transcription, although via an indirect effect of temperature induced hypoxia, is the hypoxia inducible factor HIF-1. HIF-1 protein stabilization was observed in temperate eelpout (*Zoarces viviparus*) during winter cold [35], [36]. Parallel UCP2 was up-regulated in cold adapted eelpout [25]. In addition, ROS over production in CNS caused the expressions of HIF-1 responsive genes, such as glucose transporters (GLUTs), vascular endothelial growth factor (VEGF), and erythropoietin (EPO), for supporting ATP production and facilitating oxygen supply [37]. There is considerable evidence supporting the issue of bidirectional crosstalk between mitochondrial ROS and HIF activity. These ROS may act as signaling molecules that somehow influence the regulation of the HIF pathway during hypoxia [38], [39], [40].

Although the response to cold challenge in teleost fish has been intensively studied, the molecular and physiological mechanisms and mutual relations protecting fish CNS against cold induced ROS damage are not at all understood. Therefore, particular attention should be paid on the regulatory aspects of gene transcription, involving HIF and the PPAR/UCP system. Moreover, the CNS cellular metabolism modulation should also be examined. In the present study we used the warm adapted zebrafish model, *Danio rerio*, to study the effects of acute cold exposure (from 28°C to 18°C) on fish brain. The zebrafish model is backed by a genetic database, and its applicability to study various molecular/cellular pathways and pathologies has been confirmed [41], [42]. UCP homologs in zebrafish were explored from genomic sequence analysis to transcript expressions. Specifically, we have, for the first time, measured the expression levels of UCP/PPAR, an oxidative stress parameter and several antioxidative parameters in the CNS of zebrafish upon acute cold exposure. In addition, HIF-1α protein content and the transcript levels of HIF-regulated GLUTs were quantified to test for an involvement of UCPs and HIF in modulating stress during cold exposure in fish brain.

## Results

### Phylogenetic analysis, sequence identity and gene structures of zUCPs

**Figure 1:**
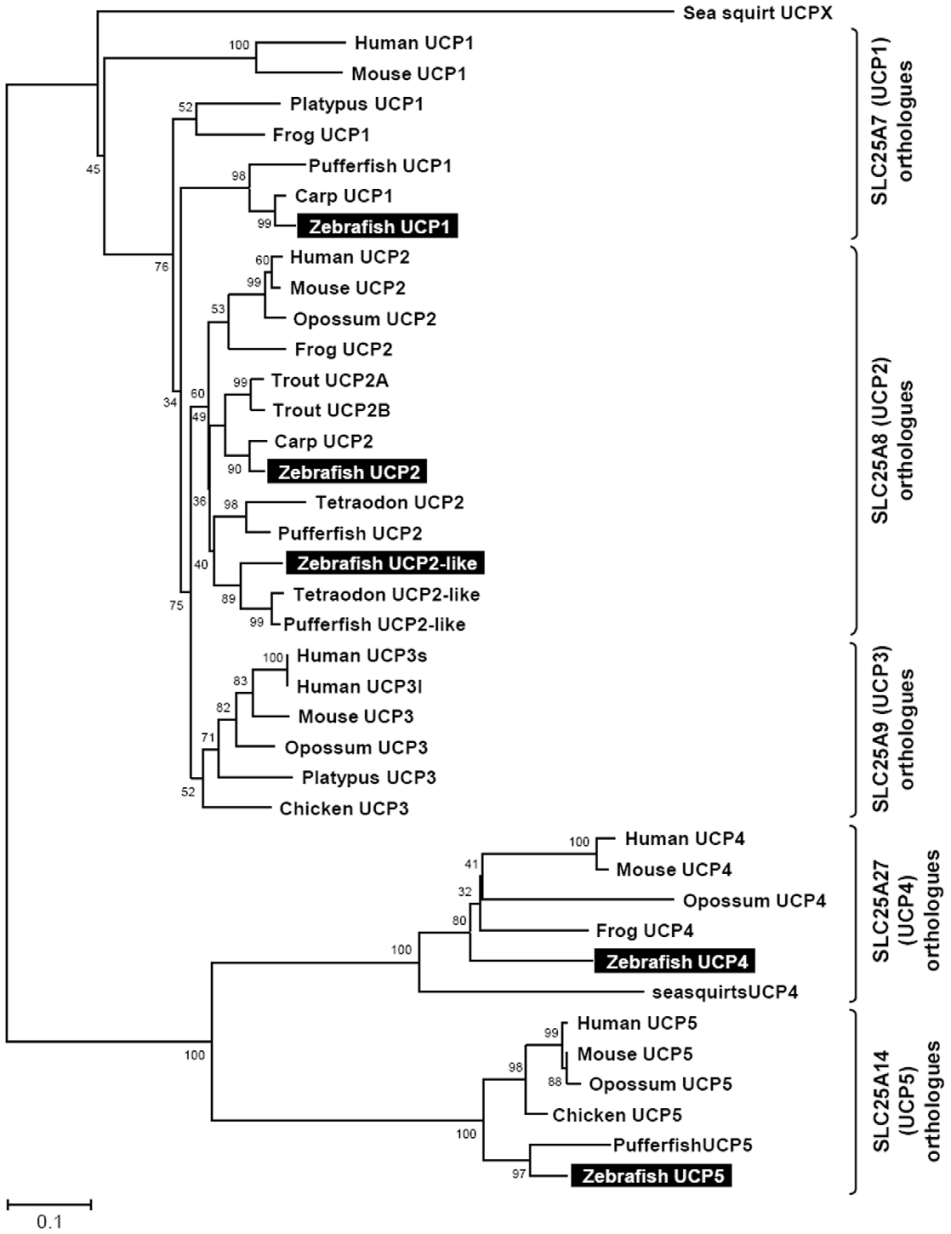
Phylogenetic analysis of UCP amino acid sequences. The putative sequences of other species and zebrafish (*Danio rerio*) were obtained from the NCBI and Ensembl databases as listed in Supporting Information S2. Consensus trees were generated using the Neighbor-joining method with the pairwise deletion gap calculating option. The results were confirmed by 1,000 bootstraps. The unit of scale bar is the number of amino acid substitutions per site.

**Figure 2:**
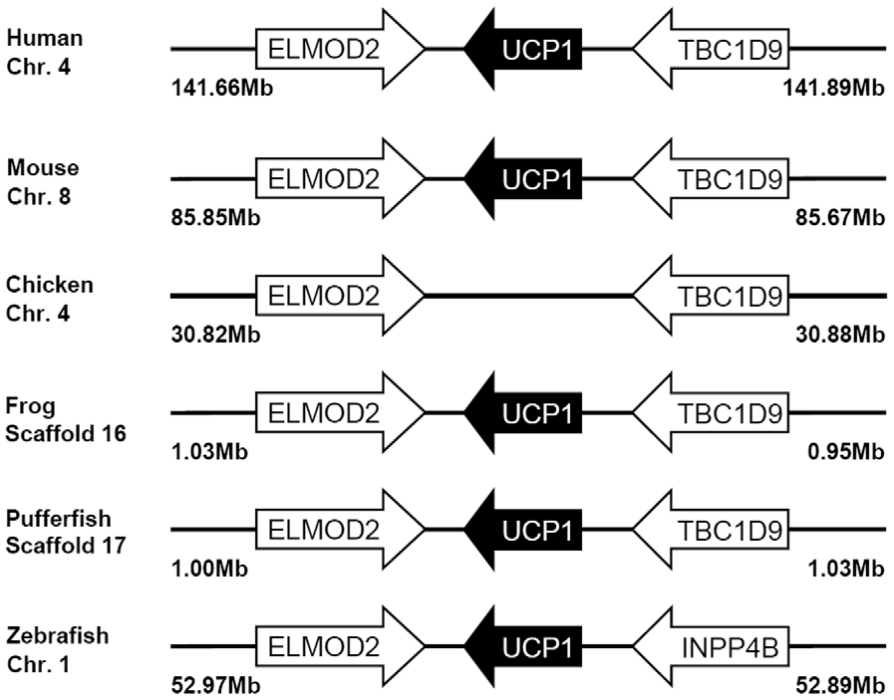
Gene structures encompassing UCP1 orthologs. The physical distance of the genomic region is indicated on both sides. Chr., the chromosome. The arrow indicates the gene with the direction. All sequences of UCP orthologs obtained from the NCBI and Ensembl database are referring to Supporting Information S2. The un-annotated proteins of *Xenopus tropicalis* and pufferfish were obtained from the Ensembl database. ENSXETT and ENSTRUT indicate the symbols of Ensembl transcript ID of *Xenopus tropicalis* and pufferfish, respectively. Those *zucp* neighboring transcripts were identified utilizing the Ensembl genome browser system. ELMOD2, ELMO/CED-12 domain containing 2; INPP4B, inositol polyphosphate-4-phosphatase, type II; TBC1D9, TBC1 domain family, member 9.

**Figure 3:**
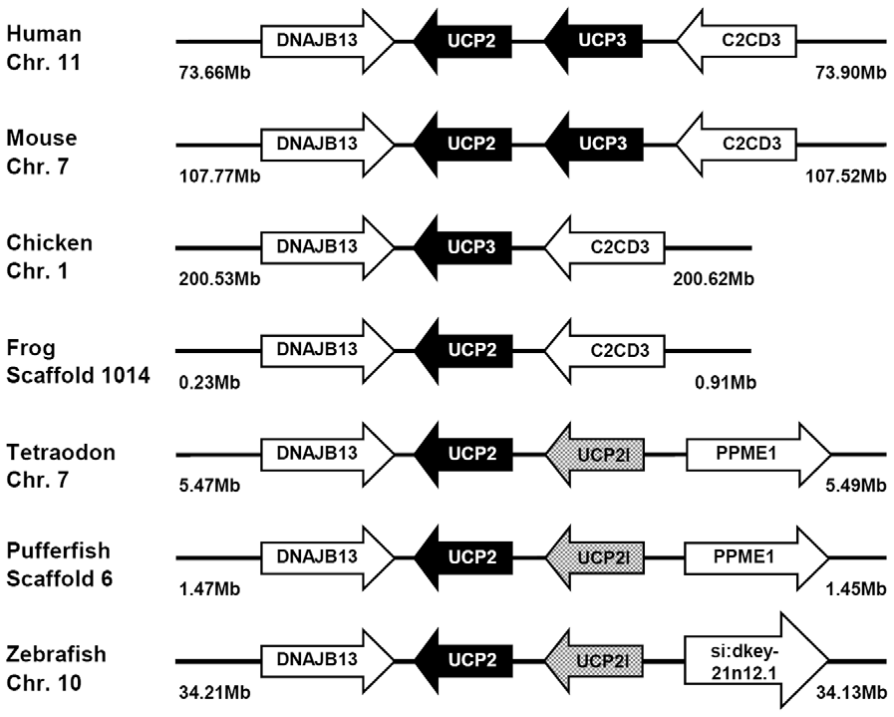
Gene structures encompassing UCP2 and UCP3 orthologs. The physical distance of the genomic region is indicated on both sides. Chr., the chromosome. The arrow indicates the gene with the direction. All sequences of UCP orthologs obtained from the NCBI and Ensembl database are referring to Supporting Information S2. The un-annotated proteins of tetraodon, and pufferfish were obtained from the Ensembl database. GSTENT and ENSTRUT indicate the symbols of Ensembl transcript ID of tetraodon, and pufferfish, respectively. Those *zucp* neighboring transcripts were identified utilizing the Ensembl genome browser system. C2CD3, C2 calcium-dependent domain containing 3; DNAJB13, DnaJ (Hsp40) homolog, subfamily B, member 13; PPME1, protein phosphatase methylesterase 1; si:dkey-21n12.1, si:dkey-21n12.1 protein.

**Figure 4:**
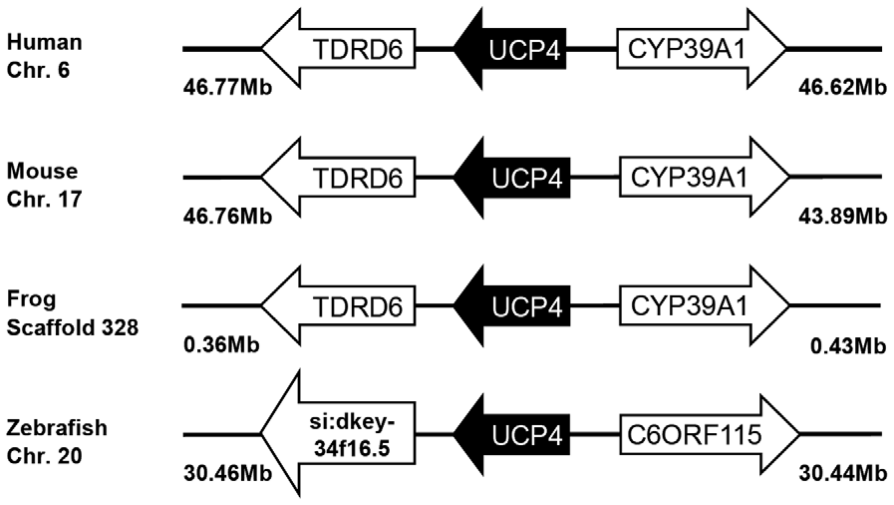
Gene structures encompassing UCP4 orthologs. The physical distance of the genomic region is indicated on both sides. Chr., the chromosome. The arrow indicates the gene with the direction. All sequences of UCP orthologs obtained from the NCBI and Ensembl database are referring to Supporting Information S2. The un-annotated protein of *Xenopus tropicalis* was obtained from the Ensembl database. ENSXETT indicates the symbols of Ensembl transcript ID of *Xenopus tropicalis*. Those *zucp* neighboring transcripts were identified utilizing the Ensembl genome browser system. C6orf115, chromosome 6 open reading frame 115; CYP39A1, cytochrome P450, family 39, subfamily A, polypeptide 1; si:dkey-34f16.5, si:dkey-34f16.5 protein; TDRD6, tudor domain containing 6.

**Figure 5:**
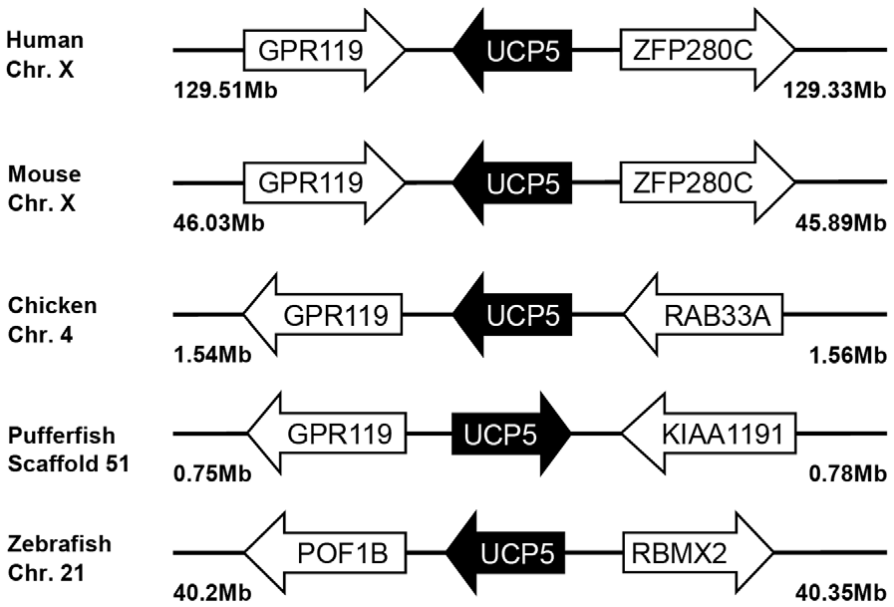
Gene structures encompassing UCP5 orthologs. The physical distance of the genomic region is indicated on both sides. Chr., the chromosome. The arrow indicates the gene with the direction. All sequences of UCP orthologs obtained from the NCBI and Ensembl database are referring to Supporting Information S2. The un-annotated protein of pufferfish was obtained from the Ensembl database. ENSTRUT indicates the symbols of Ensembl transcript ID of pufferfish. Those *zucp* neighboring transcripts were identified utilizing the Ensembl genome browser system. GPR119, G protein-coupled receptor 119; KIAA1191, KIAA1191 protein; POF1B, premature ovarian failure, 1B; RAB33A, RAB33A, member RAS oncogene family; RBMX2, RNA binding motif protein, X-linked 2; ZFP280C, zinc finger protein 280C.

Multiple sequence alignment and phylogenetic (NJ) analysis with homologues of other species clearly identified 5 members of the zUCP family which enables unambiguous identification of the zebrafish homologues (Fig. 1). The relative sequence identity between zUCPs is shown in Table 1. To further identify these *zucp* genes, comprehensive searches were performed to confirm these orthologs and determine their genomic locations (Figs. 2, 3, 4, 5). In the genome sequences of zebrafish, each isoform of *zucps* was located on different chromosome, except *zucp2* and *zucp2l*, which were located between 34.13 mega base-pairs (Mb) and 34.21 Mb on the same chromosome 10 (Fig. 3). Further, gene arrangements in the genomic regions encompassing *ucps* were compared. As shown in Figs. 2, 3, 4, 5, z*ucp* genes have their own syntenies, meaning that they co-localize adjacent to different genes than in mammals and higher ectotherms, but the genetic environment also differs in the pufferfish. Comparing the gene arrangements between each *zucp* isoform (Figs. 2, 3, 4, 5), conserved synteny of *ucp1* was found across humans, rodents, amphibians (Fig. 2). In the genome sequences of human and rodent *ucp2* and *ucp3* are located next to each other, whereas chicken and amphibians have either *ucp3* or *ucp2*, respectively (Fig. 3). Exact phylogenetic analysis, revealed *ucp2-like (ucp2l)* genes are newly annotated and only found in fish (Fig. 3). In addition, gene arrangements of *ucp2* and *ucp2l* in the genomic regions encompassing these fish isoforms were compared. These homologues in zebrafish, tetradon and pufferfish are located adjacent to *ucp2* on the same chromosome (Fig. 3). Phylogenetic inference grouped SLC25A27 (UCP4) and SLC25A14 (UCP5) into another root from other members (Fig. 1). The syntenies found around *ucp4* and *ucp5* between humans and rodents are not conserved across chicken, amphibians, zebrafish, tetraodon (*Tetraodon nigroviridis*), and pufferfish (*Takifugu rubripes*) (Figs. 4, 5).

**Table 1:** Identities (in percent) of amino acid sequences among the identified zebrafish UCP (zUCP) isoforms.

Identity(%)	*zucp1*	*zucp2*	*zucp2l*	*zucp4*	*zucp5*
***zucp1***	-	72	46	33	33
***zucp2***		-	50	32	34
***zucp2l***			-	23	22
***zucp4***				-	36
***zucp5***					-

### *zucp* mRNA expressions in zebrafish at 28°C

**Figure 6:**
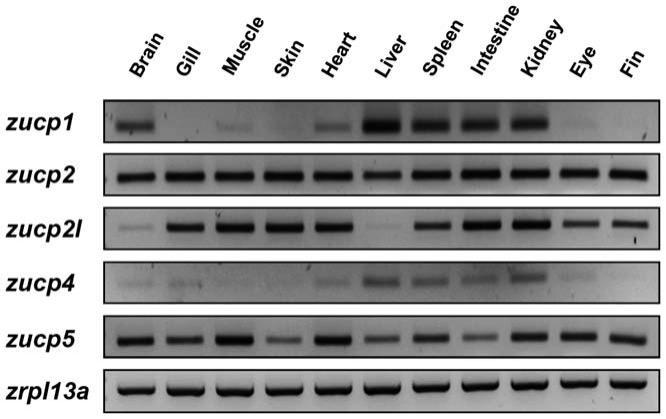
*zucp* mRNA expressions in various tissues of zebrafish. Isoform-specific primer sets were used for RT-PCR analysis. *rpl13a* was used as the internal control.

Expression of *zucp* mRNAs was evaluated by RT-PCR in different zebrafish tissues (Fig. 6). All isoforms were expressed in brain, heart, spleen, intestinal wall and kidney. mRNA expressions of the z*ucp2* and *zucp5* isoforms were ubiquitously expressed in all tissues. Contrary, mRNA levels of z*ucp4* were low compared to other isoforms in all tissues.

### Localization of *zucp* mRNAs in zebrafish brain at 28°C

**Figure 7:**
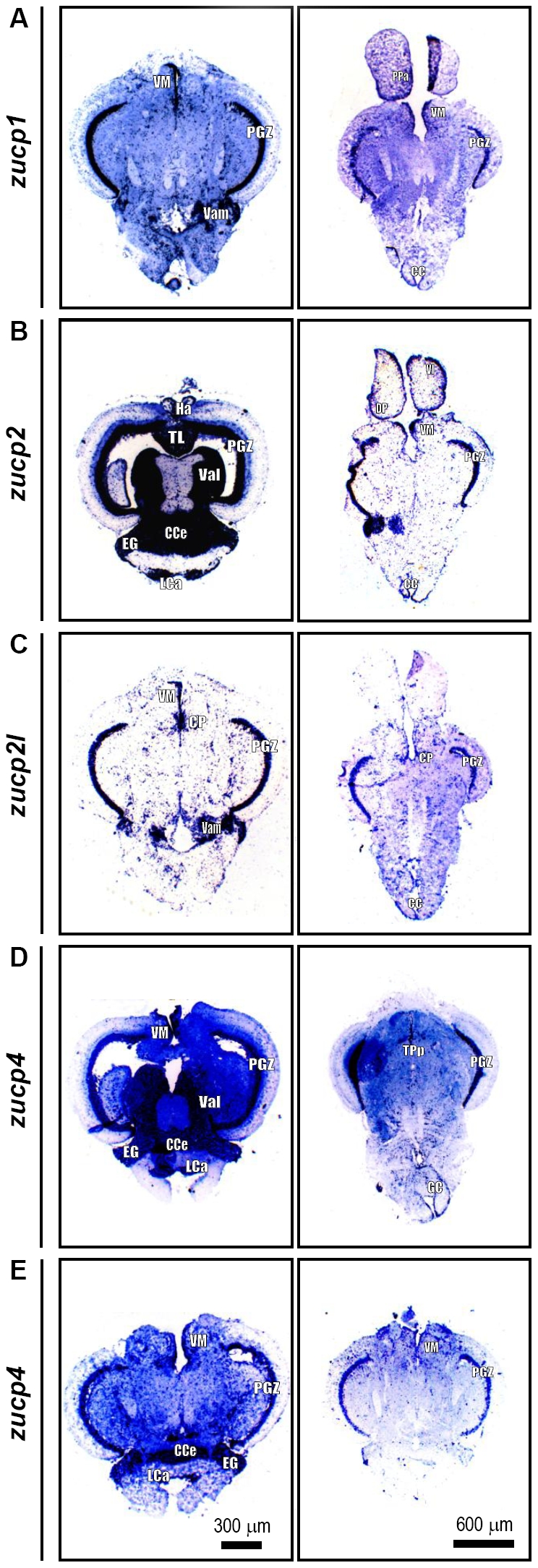
mRNA *in situ* hybridization of *zucp* paralogs in brains' cross section of adult zebrafish. All the five *zucps* were observed in diencephalon, mescencephalon, and cerebellum. In addition, except the *zucp5*, *zucp1, -2, -2l, -4* were all expressed in medulla oblongata. Besides, *zucp2* mRNA also distributed in telencephalon. Detailed mRNA expression patterns of *zucps* in zebrafish brain were listed in Table 2. CC, cerebellar crest; CCe, cerebellar corpus; CP, central posterior thalamic nucleus; DP, dorsal posterior thalamic nucleus; EG, granular eminence; Ha, habenula; LCa, caudal lobe of cerebellum; PGZ, periventricular gray zone of optic tectum; PPa, parvocellular preoptic nucleus; TL, longitudinal torus; TPp, periventricular nucleus of posterior tuberculum; Val, lateral division of valvula cerebelli; Vam, valvula crebelli; Vl, lateral nucleus of ventral telecephalic area; VM, ventromedial thalamic nucleus.

In subsequent experiments, specific RNA probes were designed to conduct *in situ* hybridization of the 5 *zucp* isoforms in different horizontal axis cryo-sections of zebrafish brain. As shown in Fig. 7A, *zucp1* was predominately localized in the anterior part of the medial division of the cerebellar crest (CC), the valvula crebelli (Vam), the parvocellular preoptic nucleus (PPa), the periventricular gray zone of the optic tectum (PGZ) and the ventromedial thalamic nucleus (VM). mRNA of *zucp2* was strongly stained in CC, caudal lobe of the cerebellum (LCa), the cerebellar corpus (CCe), the dorsal posterior thalamic nucleus (DP), the granular eminence (EG), the habenula (Ha), the lateral nucleus of the ventral telecephalic area (Vl), the longitudinal torus (TL), the lateral division of the valvula cerebelli (Val), PGZ and VM (Fig. 7B). Positive signals for *zucp2l* were observed in the central posterior thalamic nucleus (CP), CC, PGZ, Vam and VM (Fig. 7C). Further, expressions of *zucp4* in zebrafish brains were found in CC, CCe, EG, LCa, PGZ, periventricular nucleus of posterior tuberculum (TPp), Val and VM (Fig. 7D). Furthermore, *zucp5* was localized in brain areas of CCe, EG, LCa, PGZ and VM (Fig. 7E). *In situ* hybrization indicated all *zucp* homologues to be expressed in PGZ and VM regions under control conditions (28°C).

**Table 2:** Summary of the detailed *zucps* mRNA expression patterns in zebrafish brain.

	*zucp1*	*zucp2*	*zucp2l*	*zucp4*	*zucp5*
***Telencephalon***		Vl			
***Diencephalon***					
- Area praeoptica	PPa				
- Epithalamus		Ha			
- Dorsal thalamus		DP	CP		
- Ventral thalamus	VM	VM	VM	VM	VM
- Posterior tuberculum				TPp	
***Mescencephalon***					
- tectum opticum	PGZ	PGZ, TL	PGZ	PGZ	PGZ
***Cerebellum***					
- Vestibulolateralis lobe		EG, LCa		EG, LCa	EG, LCa
- Corpus cerebelli		CCe		CCe	CCe
- Valvula cerebelli	Vam	Val	Vam	Val	
***Medulla oblongata***	CC	CC	CC	CC	

CC, cerebellar crest; CCe, cerebellar corpus; CP, central posterior thalamic nucleus; DP, dorsal posterior thalamic nucleus; EG, granular eminence; Ha, habenula; LCa, caudal lobe of cerebellum; PGZ, periventricular gray zone of optic tectum; PPa, parvocellular preoptic nucleus; TL, longitudinal torus; TPp, periventricular nucleus of posterior tuberculum; Val, lateral division of valvula cerebelli; Vam, valvula crebelli; Vl, lateral nucleus of ventral telecephalic area; VM, ventromedial thalamic nucleus.

### Effects of cold acclimation on mRNA expressions patterns of *zucps* in zebrafish brain

**Figure 8:**
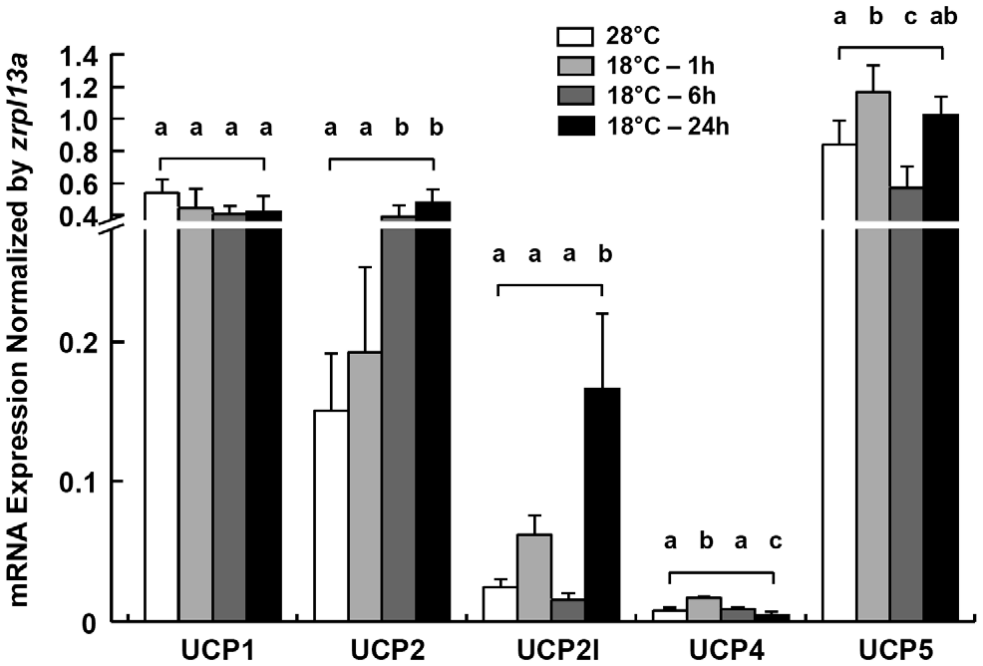
UCP expressions in zebrafish brain. qPCR analysis of 5 *zucp* isoforms in brains of zebrafish at 28°C and 1, 6 or 24 h after transfer to 18°C. *rpl13a* was used as the internal control. Data are presented as mean ± SD (*N* = 4). Different letters indicate significant differences between treatments (one-way ANOVA, Tukey's pairwise comparison, *p*<0.05).

The time-course changes of *zucp*s' mRNA expression in brain of zebrafish transferred from 28°C to 18°C at different times of cooling up to 24 h are shown in Fig. 8, with ribosomal protein L13a (*zrpl13a*) as house keeping gene. At 28°C control temperature, *zucp1* and *zucp5* were stronger expressed in brains compared to *zucp2l* and *zucp4* (Fig. 8). Furthermore, *zucp5* exhibited the highest mRNA levels in brains of zebrafish at control temperature and was about 80-fold higher expressed than *zucp4*. During cold-shock (1 h after transfer) and a 6 and 24 h of cold acclimation, *zucp1* transcript expression remained invariably high without any change, whereas expression of *zucp2* increased significantly by about 3-fold at 6 and 24 h of cold exposure compared to control group. In addition, the *zucp2l* mRNA was significantly induced after 24 h at 18°C. Expression of *zucp4* transcript in brain was always very low, but was rapidly up-regulated within 1 h of acute cold shock, whereafter expression returned to control level at 6 h cooling at 18°C. After 24 h at 18°C, *zucp4* transcript levels were lower than in control fish brain at 28°C. The time profile of brain *zucp5* mRNA expression resembled that of *zucp2l*. However, the expression level was generally higher than *zucp2l* and up-regulation of *zucp5* transcript 1 h after transfer to 18°C was significant. At 6 h of cold exposure the expression declined to below control level (28°C), but recovered again reaching control and 1 h cold exposure levels at 24 h of cold exposure. These results in zebrafish brain demonstrate that mRNA expressions of four *zucp* isoforms are significantly affected by cold stress, except the *zucp1* isoform.

### Changes of oxidative stress parameters in zebrafish brain

**Figure 9:**
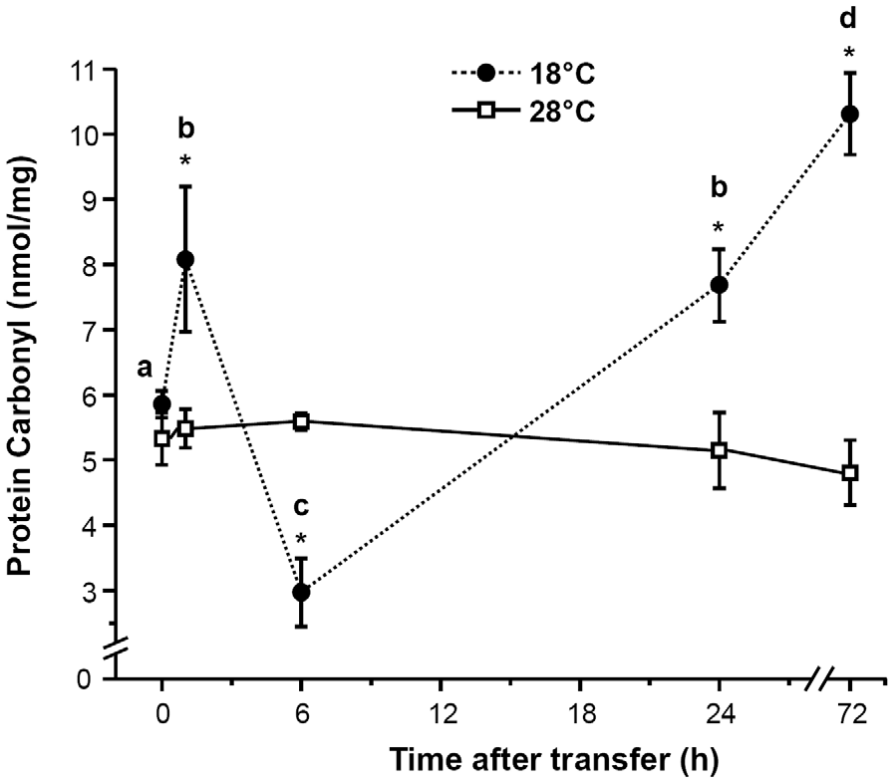
Protein carbonyl contents in zebrafish brain. Time-course of changes in the level of protein carbonyl in the brains of zebrafish that was transferred from 28°C (0 h) to 18°C. The carbonyl contents were measured by detecting their dinitrophenylhydrazones (DNP). The solid line represents the 28°C control group, and the dashed line represents the 18°C treatment group. Data are presented as means ± SD. (*N* = 6). *Indicates a significant difference from the respective control in 28°C (*p*<0.05). Different letters indicate significant differences (*p*<0.05) among sampling times in fish transferred to 18°C.

**Figure 10:**
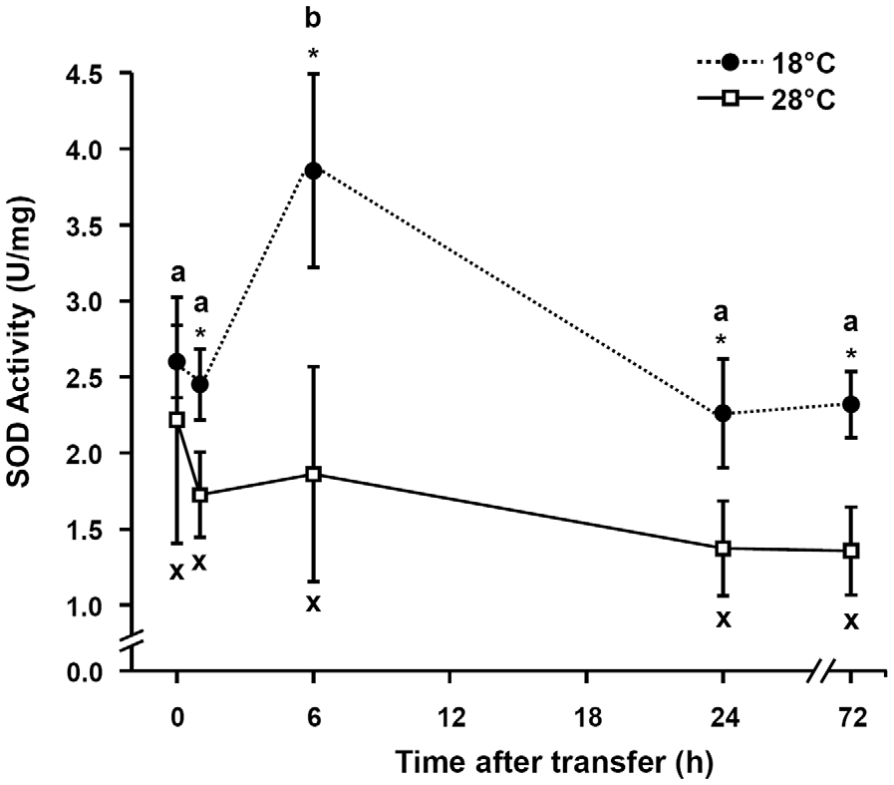
SOD expressions in zebrafish brain. Time-course of changes in the specific activity of superoxide dismutase (SOD) in the brains of zebrafish that were transferred from 28°C (0 h) to 18°C. The SOD activities were determined with xanthine/xanthine oxidase (XOD) system. The solid line represents the 28°C control group, and the dashed line represents the 18°C treatment group. Data are presented as means± SD. (*N* = 6). *Indicates a significant difference from the respective control in 28°C (*p*<0.05). Different letters indicate significant differences (*p*<0.05) among sampling times in fish transferred to 18°C.

Proteins are a major target of oxygen free radicals and other reactive species during several forms of stresses. Fig. 9 presents the time-course of the content of protein carbonyls in zebrafish brain at different time points after transfer from 28°C to 18°C. Protein carbonyl content increased significantly by about 38% (*p*<0.05) within the first hour of acute cold shock, but declined dramatically to very low levels after 6 h of cold exposure. Subsequently, the brain carbonyl content increased again and was significantly above control level at 24 h and 72 h of cold acclimation. The time pattern of the specific SOD activity during cold exposure (Fig. 10) remained constant compared to the control group within the first hour after transfer to 18°C but increased to about 160% after 6 h cold exposure. On prolonged exposure to 18°C, SOD activity decreased again to control level.

**Figure 11:**
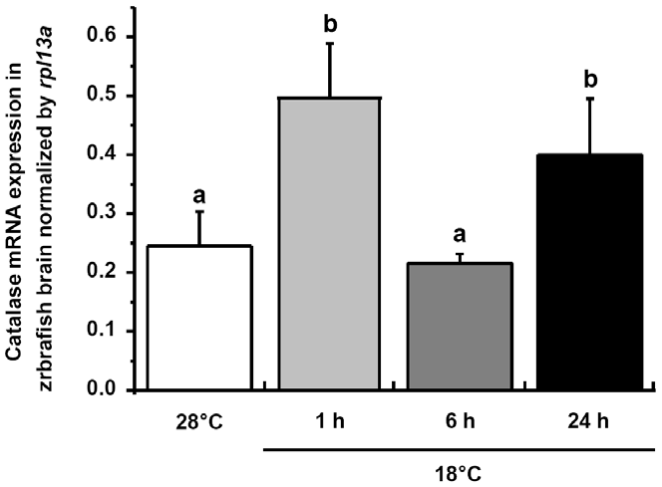
Catalase expressions in zebrafish brain. qPCR analysis of catalase (*zcat*) mRNA expressions in brains of zebrafish at 28°C and 1, 6 or 24 h after transfer to 18°C. *rpl13a* was used as the internal control. Data are presented as mean ± SD (*N* = 4). Different letters indicate significant differences between treatments (one-way ANOVA, Tukey's pairwise comparison, *p*<0.05).

Zebrafish catalase (*zcat*) transcript expression in brain increased rapidly during the first hour of cold exposure (to about 190%, Fig. 11), whereas expression levels after 6 h cold exposure were back to control level. At 24 h following cold transfer, *zcat* mRNA expression was again elevated above the 6 h expression level.

**Figure 12:**
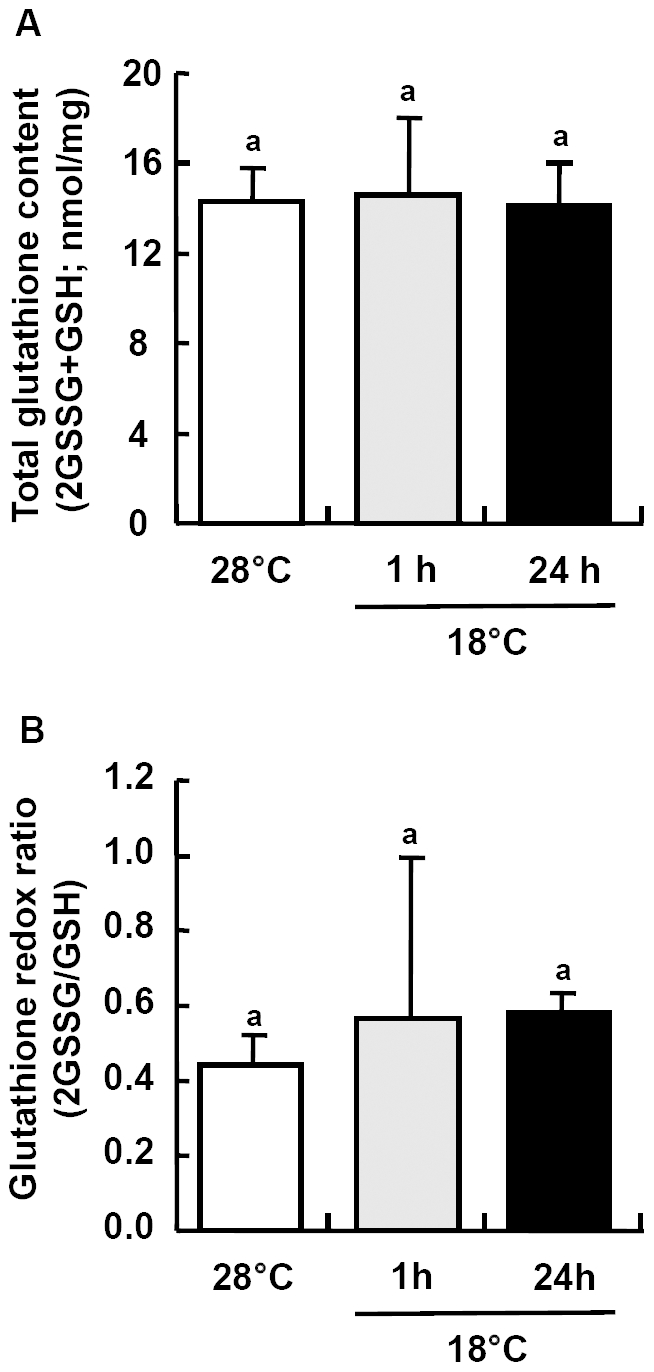
Expressions of glutathione in zebrafish brain. Total glutathione content (GSx) (A) and GSSG/GSH ratio (B) in zebrafish brains at 28°C and 1 h or 24 h after transfer to 18°C. Data are presented as mean ± SD (*N* = 4). Different letters indicate significant differences between treatments (one-way ANOVA, Tukey's pairwise comparison, *p*<0.05).

Neither the ratio of oxidized (GSSG) to reduced (GSH) glutathione, nor the total glutathione content in zebrafish brain changed significantly between controls and the cold exposed group at the investigated time points (Fig. 12A, B), indicating that the thiol reduction potential is maintained constant in the brains of zebrafish that had been exposed to acute cold stress.

### mRNA expression of peroxisome proliferator-activated receptors (PPARs)

**Figure 13:**
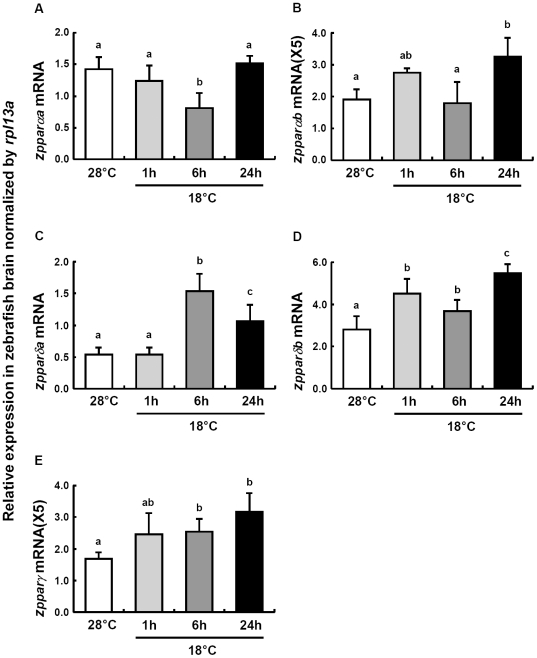
PPAR expressions in zebrafish brain. qPCR analysis of different types and isoforms of *zppar* mRNA expressions in zebrafish brains at 28°C and 1, 6 or 24 h after transfer to 18°C. (A) *zpparαa* mRNA, (B) *zpparαb* mRNA, (C) *zpparδa* mRNA, (D) *zpparδb* mRNA, (E) *zpparγ* mRNA. *rpl13a* was used as the internal control. Data are presented as mean ± SD (*N* = 4). Different letters indicate significant differences between treatments (one-way ANOVA, Tukey's pairwise comparison, *p*<0.05).

Zebrafish carry five *ppar* homologues, and all of them are expressed in brain (Fig. 13A–E). *zpparαa* transcript levels in brain were significantly decreased after 6 h of cold stress (Fig. 13A). By contrast, mRNA levels of *zpparαb* were up-regulated after 1 and 24 h in the cold, whereas 6 h post-transfer transcription was at control level (Fig. 13B). The mRNA expression of *zpparδa* was greatly up-regulated after 6 h and then decreased slightly to levels still above the 28°C controls at 24 h of cold acclimation (Fig. 13C). Across all 5 PPAR isoforms in zebrafish brain, *zpparδb* and *zpparγ* displayed the highest and lowest mRNA expression, respectively (Fig. 13D, E). The profile of *zpparδb* and *zpparγ* mRNA expression in cold exposed fish were similar to that of *zpparαb* (Fig. 13B, D, E), exhibiting a gradual increase throughout the time of experimental cold exposure.

### Effect of cold stress on hypoxic signaling: Hypoxia inducible factor (HIF-1α) and Glucose transporters (GLUTs)

**Figure 14:**
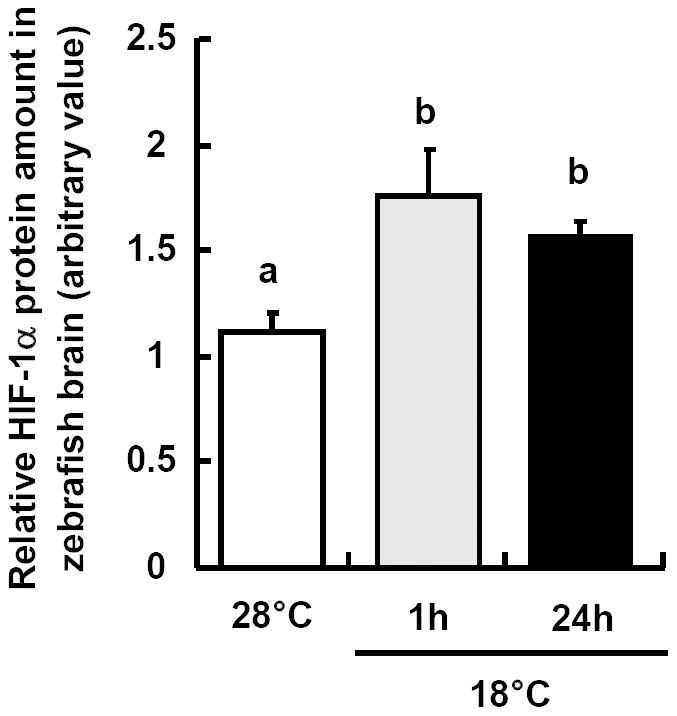
HIF-1α expressions in zebrafish brain. HIF-1α protein relative amounts in brains of zebrafish at 28°C and 1 or 24 h after transfer to 18°C. Protein amounts were determined by densitometry of the bands in Western blotting membranes. Data are presented as mean ± SD (*N* = 5). Different letters indicate significant differences between treatments (one-way ANOVA, Tukey's pairwise comparison, *p*<0.05).

**Figure 15:**
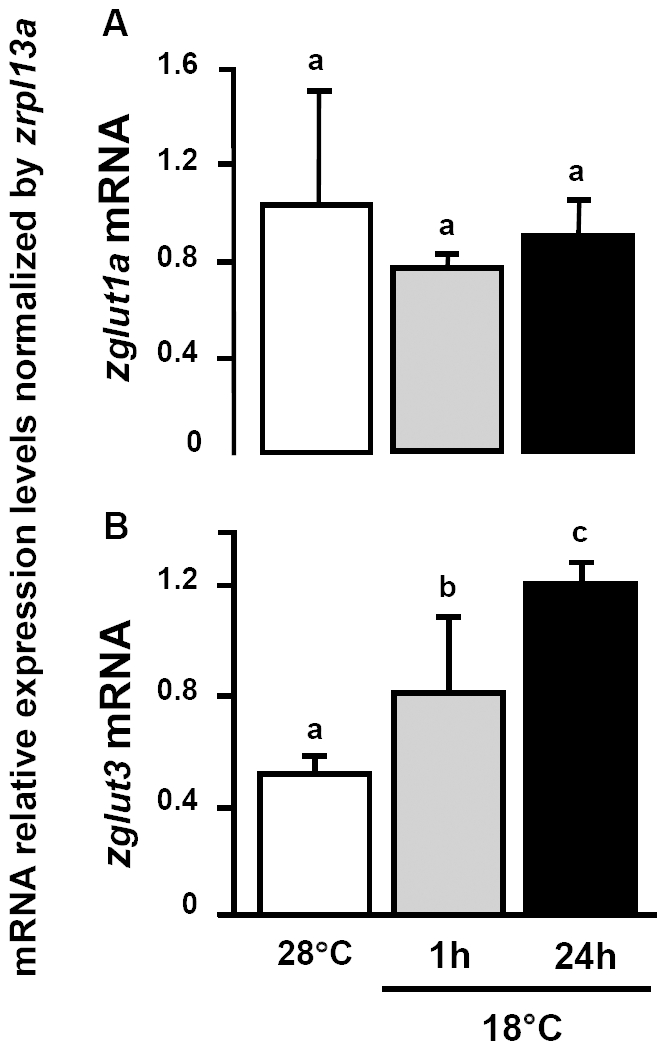
Expressions of GLUT in zebrafish brain. Comparison of *zglut1a* (A) and *zglut3* (B) transcripts (by qPCR) in adult zebrafish brains at 28°C and 1 or 24 h after transfer to 18°C. Data are presented as the mean ± SD (*N* = 4). Different letters indicate significant differences between treatments (one-way ANOVA, Tukey's pairwise comparison, *p*<0.05).

Western blot analysis with antibodies against the fish HIF-1α subunit demonstrates the stabilization of the protein in 1 and 24 h cold exposed zebrafish brain compared to control fish. The levels of HIF-1α protein subunit increased to about 150% in cold exposed fish brain at both time points (Fig. 14). Both glucose transporters GLUT1 and GLUT3 are HIF-1 target genes [43]. qPCR analysis revealed no significant differences in mRNA expression of *zglut1a* in brains of control fish (28°C) and 18°C exposed fish (Fig. 15A). However, *zglut3* transcripts were up-regulated by about 160% and 260% of controls 1 and 24 h after transfer of the fish to the 18°C environment, respectively (Fig. 15B).

## Discussion

To our knowledge, this is the first time that all paralogs of the UCP family in a species, the zebrafish, were investigated within a study across all organs. All the *zucp* isoforms were expressed in every region of the brain, and only the *zucp1* mRNA expression was not induced during cold exposure.

An analysis of UCP homologue evolution by Sokolova and Sokolov [28] starting with the invertebrate UCP isoforms suggests that the divergence of UCPs is an early evolutionary event, which explains the functional diversity of this protein family in vertebrates and mammals. The authors further observed the transcript of UCP5 in different tissues (e.g. gills, muscles, hepatopancrease) of eastern oyster (*Crassostrea virginica*) varied under hypoxia-reoxygenation stress, cadmium exposure and temperature fluctuations [44]. Saito and colleagues also proposed that the NST function of mammalian UCP1 was acquired through positive Darwinian selection and, further, suggested that vertebrate UCP1-3 acquired much of their diversity through two rounds of gene duplication. The proto-UCP first duplicated into UCP1 and ancestral UCP2/UCP3, and then the second gene duplication produced UCP2 and UCP3 which is estimated to be 420 million years ago [27]. Fig. 3 documents this duplication of UCP2 (*ucp2* and *ucp2l*) in teleosts. The duplication occurred in the chromosome of fish which parallels the loci of UCP2 and UCP3 in mammals. However, amino-acid identity between *zucp2* and *zucp1* (72%) is higher than between *zucp2* and *zucp2l* (50%) (Table 1), and, moreover, *zucp2l* shows a different expression pattern than human *ucp3* which is mostly restricted to skeletal muscle (Fig. 6; [45]). Taken together, the great dissimilarity in the UCPs' alignments between fish and mammals are suggestive of differences in physiological functions and uncoupling activities, and various roles of UCP paralogs have been discussed beyond their function in BAT (brown adipose tissue) in mammals [46]. Ectothermic fish cope with wide fluctuations of habitat temperature and their metabolic rates follow the changing thermal patterns. Subtle and transient change in environmental temperature do not cause changes of mitochondrial densities as found during seasonal or climatic thermal adaptations [47], [48], but may met by the modulating effect of UCPs on both phosphorylation rates and ROS formation at high temperature. According to relative studies in common carp, UCP1 mRNA levels in brain were up-regulated obviously in response to 7–10 days of cold acclimation [23]. In this study, four *zucps* in the zebrafish brain showed increased expression upon acute cold exposure (Fig. 8), suggesting the divergent roles of the 4 *zucp* isoforms in metabolic balance in fish brain.

The ubiquitious UCP homologue distribution patterns in all parts of the brain, especially PGZ and the cerebellum suggest that they may participate in neuronal circuits (in the PGZ of the optical tectum) and neuroendocrine functions for metabolic homeostasis [23], [49], [50], [51]. Similar distribution pattern in PGZ of optic tectum as carp UCP1 implied zUCPs may also be involved in the control of sensory function [23]. This could possibly imply that the sensorimotor pathway in the brain of teleost fish is activated by fluctuations of the ambient temperature [52]. Under these circumstances, mitochondrial respiration rates and oxygen diffusion and delivery to central organs such as the CNS undergo rapid change (slower in cooling, faster during warming), and perhaps different zUCPs might serve to adjust mitochondrial function during sudden warming and cooling, or other stress conditions. ROS and lipid peroxidation products could, indeed, play a role in UCP induction during the onset of thermal stress in fish. Thus, the cellular biomarkers for protein oxidation increased dramatically in brains of cold exposed zebrafish in our experiments. At the same time, SOD activity was up-regulated already starting after 1 h at 18°C counteracting uncontrolled ROS formation during cooling, so that GSH concentration and thiol reduction potential remained constant, although highly variable between samples.

**Figure 16:**
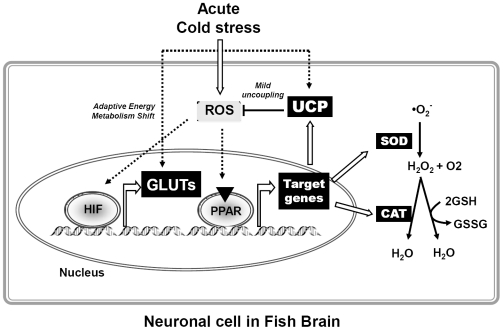
Proposed responses in zebrafish brain upon cold stress. Schematic diagram illustrating physiological pathways postulated to become activated by cold-induced oxidative stress in zebrafish brain. Cold exposure increases ROS levels in zebrafish brain, which activate the PPARs. Activated PPARs may enhance the expression of their target genes, including UCPs, SOD and CAT. Increased SOD activity and *cat* expression will reduce superoxide more rapidly to H_2_O, thus slowing down the ROS accumulation. In addition, activated UCPs may reduce the mitochondrial ROS levels through mild uncoupling. Moreover, the cold-induced ROS may act as signaling molecules that influence HIF activity and further modulate the expression of HIF targeted genes, such as GLUTs, which could subsequently mediate an adaptive shift in energy metabolism. Open arrows indicate according relationships that prove in the present study, and dotted arrows indicate inferred relationships infer from this study.

Several recent studies investigated activation of glycolysis during brain hypoxia exposure. Increased glucose uptake, glycolysis rates and cellular lactate levels were observed to compensate for the reduced mitochondrial ATP production resulting from expression of UCP [53]. The adaptive shift in metabolism and neuroprotective mechanisms is crucial for satisfying the brains high energy demand during hypoxia [53]. Our study is in agreement with these findings, as we found that HIF-1α protein level was rapidly increased compared to control and was maintained above control for up to 24 h in the cold exposed zebrafish. This suggests that brain hypoxia sets on in zebrafish within 1 h of acute cold stress. As shown in our concept in Fig. 16, we inferred that the excess of ROS or lipid peroxidation products accumulating in fish brain during cold exposure up-regulate some *ucp* genes. Further, under cellular hypoxia, HIF-1α protein stabilizes and induces a hypoxic response ensuring increased glucose supply to the brain [54] by up-regulating the HIF-target *zglut3* expression upon cold exposure (Fig. 15B). Based on GLUT relevant studies in mammals, GLUT3 is considered neuron-specific [55]. Thus, HIF-1 mediated up-regulation of the expression of *zglut3* could support especially neurons with additional glucose demand during cold stress. In contrast, expression of *zglut1a* mRNA was not changed in 18°C acute cooling situation (Fig. 15A), suggesting that this GLUT isoform, which in the mammalian brain is mainly expressed in glial cells [55], is not requested for additional carbohydrate supply to brain cells upon cold stress. Therefore, during acute cold stress, supply of neurons with glucose appears to be more required than for glial cells.

The transactivation mechanisms of PPAR-induced neuroprotection including oxidative stress modulation and anti-inflammatory effect have been postulated in recent studies [33]. In brains of zebrafish, the various mRNA expression patterns of diverse types/isoforms of PPAR represent different responses after cold exposure (Fig. 13). In addition, the mRNA expression patterns of *zucp2* and *zucp2l/zucp5* are similar to that of *zpparγ* and *zpparδb,* respectively (Figs. 8, 13D, E), which implied that different *zppar* homologs may induce the expression of respective *zucps*. Apart from the control of UCPs gene expression, to date, emerging evidence suggests that mammalian PPARs participate in the modulation of expression of antioxidant enzymes, including SOD and catalase [56], [57], [58]. SOD1 was reported to be activated partly through the peroxisome proliferator response element (PPRE) in its promoter [59]. In the study of PPARδ knockout in adult mouse heart, PPARδ has been proved to regulate both SOD1 and SOD2 expression [60]. In the present study, the expression pattern of SOD activity in brain parallelsed that of *zpparδa*; both of them showed a dramatic increase and decrease after 6 and 24 h post-transfer, respectively (Figs. 10, 13C). Furthermore, the PPRE has been recently identified in the promoter area for the catalase gene [56] and catalase has been proposed as one of the target enzymes of PPARα in rat liver [58]. In brains of zebrafish, *zcat* and *zpparαb* mRNA expressions also showed parallel pattern, which was upregulated after 1 and 24 h (Figs. 11, 13B), suggesting that also in the zebrafish brain PPAR activation may activate catalase expression.

The present study is the first to indicate that PPAR activation pathways may control the superoxide accumulation in fish brain by enhancing the activity/expression of antioxidant enzymes such as SOD and catalase. Simultaneous up-regulation of PPAR and UCPs suggest a direct coupling of theses pathways which could lower mitochondrial ROS levels by mild uncoupling. These integrated studies in ectotherms provide novel insights into an antioxidant mechanism in brain under cold disturbance through PPAR pathways, UCP activations, HIF regulations and changes in metabolism. The rapid physiological adaptation in brain may enable ectothermic fish to cope with rigorous temperature drops without immediately incurring death.

## Materials and Methods

### Animals

Adult zebrafish (*D. rerio*, body weight 0.5∼0.6 g) brood stocks at the Institute of Cellular and Organismic Biology, Academia Sinica (Taipei, Taiwan) were kept in local tap water at 28°C under a 14-h light: 10-h dark photoperiod. During experiment, a group of ten zebrafish was kept in 1∶1 ratio of female and male within a 30-L flow-through circular test aquarium equipped with an activated filter carbon filtration system. Each of the test aquaria in the flow-through system was individually delivered by a mixture of water fitted with a cooling device to maintain the constant temperature (18±1°C). Experimental protocols were approved by the Academia Sinica Institutional Animal Care and Utilization Committee (approval no.: RFIZOOHP220782).

### Acclimation experiments

Zebrafish acclimated to 28°C were directly transferred to 18°C and acclimated for defined time intervals (between 1 h and 24/72 h) according to previous studies [61], [62]. Fish were fed during acclimation. Adult zebrafish were fed three times per day with dry food (Hai Feng, Nantou, Taiwan) corresponding to 3% of average body weight and once per day with Artemia. At the end of the acclimation period, zebrafish were euthanized with buffered MS-222 (Ethyl 3-aminobenzoate methanesulfonate salt, 0.03%). Afterwards, fish were dissected on ice and the organs sampled for total RNA, protein extraction and oxidative stress parameter analysis in liquid nitrogen. Fish were always sacrificed during the same time between 11:00 AM to 1:00 PM in order to normalize effects of circadian rhythms on physiology.

### Preparation of mRNA

The total RNA was extracted by homogenizing zebrafish tissues (brain, gill, muscle, skin, heart, liver, spleen, intestine, kidney, eye, and fin) in Trizol Reagent (Invitrogen, Carlsbad, CA, USA) and DNA contamination removed with DNase I (Promega, Madison, WI, USA). The mRNA for the RT-PCR was obtained with a QuickPrep Micro mRNA Purification Kit (Amersham Pharmacia, Piscataway, NJ, USA) according to the supplier protocol. The amount of mRNA was determined by spectrophotometry (ND-1000, NanoDrop Technol, Wilmington, DE), and the mRNA quality was checked by running electrophoresis in RNA denatured gels. All mRNA pellets were stored at -20°C.

### Phylogenetic and genomic analysis

The full-length coding sequences of known zebrafish UCP homologs were obtained from the GenBank. For identification and the phylogenetic analysis of UCP candidates, the deduced amino acid sequences of zebrafish UCPs were aligned together with all known UCP protein sequences available in public databases using ClustalX (National Center for Biotechnology Information, Bethesda, MD, USA) and subjected to phylogenetic inferences using the Neighbor-joining (NJ) method. One thousand bootstrap replicate analyses were carried out with Mega4.0. Physical gene maps of verified *ucp* loci were scaled, based on assemblies of the Ensembl Genome Browser. Genes located up- and downstream of *ucp* genes in these loci were blasted against mammalian genomes to determine the highest score.

The zebrafish UCPs, PPARs and catalase sequences obtained from GenBank were used to design PCR primers for real-time reverse transcriptase PCR and to generate probes for *in situ* hybridization. Primers are summarized in Supporting Information S1.

### Brain sections *in situ* hybridization

Fresh zebrafish brains were fixed with 4% paraformaldehyde at 4°C for 3 h, and then gradually immersed in PBS (0.09% NaCl in 0.1 M phosphate buffer) containing different concentrations of sucrose of 5%, 10%, and 20% at 4°C. Samples were soaked in a mixed PBS solution (OCT compound: 20% sucrose 1∶ 2) over night and embedded with OCT compound-embedding medium (Sakura, Tokyo, Japan) at -20°C. Cryostat (CM 1900, Leica, Heidelberg, Germany) sections of 10 µm were applied to poly-L-lysine-coated slides (Erie, Hooksett, NH, USA).

For *in situ* hybridization, digoxigenin (DIG)-labeled (Perkin-Elmer, Boston, MA, USA) RNA probes were synthesized by *in vitro* transcription with SP6 RNA polymerase (Takara, Shiga, Japan). Brain *in situ* hybridization was performed as previously described [63] and conducted with the nitro blue tetrazolium (NBT) and 5-bromo-4-chloro-3-indolyl phosphate (BCIP) system.

### Real-time quantitative (q)PCR

Total RNA was extracted and reverse-transcribed from adult brains of zebrafish as described above. The mRNA expressions of target genes were measured by qPCR with the Roche LightCycler® 480 System (Roche Applied Science, Mannheim, Germany). Primers for all genes were designed (Supporting Information S1) using Primer Express software (vers. 2.0, Applied Biosystems). PCRs contained 40 ng of cDNA, 50 nM of each primer, and the LightCycler® 480 SYBR Green I Master (Roche) in a final volume of 10 µl. All qPCR reactions were performed as follows: 1 cycle of 50°C for 2 min and 95°C for 10 min, followed by 45 cycles of 95°C for 15 sec and 60°C for 1 min (the standard annealing temperature of all primers). PCR products were subjected to a melting-curve analysis, and representative samples were separated by electrophoresis to verify that only a single product was present. Control reactions were conducted with sterile water to determine signal background and DNA contamination. The standard curve of each gene was confirmed to be in a linear range while ribosomal protein L13a (RPL13A) was selected as a reference gene [64].

### Protein carbonyl contents measurement

Carbonyl groups were measured as indication for oxidative damage of proteins. Brains were disrupted in cold homogenization buffer (100 mM imidazole, 5 mM EDTA, 200 mM sucrose, and 0.1% sodium deoxycholate, pH 7.6). In order to avoid interference of high concentration of nucleic acid which in some tissue contribute to an over estimation of the carbonyl contents, we incubated the brain homogenates in a final concentration of 1% streptomycin sulfate for 30 min at room temperature and removed the nucleic acid precipitates by centrifuging at 6000 g for 10 min at 4°C. Brain protein carbonyls were measured using the OxiSelect Protein Carbonyl ELISA kit (Cell Biolabs, San Diego, CA, USA) following the manufacturer's protocol. The contents of protein carbonyls were measured at 450 nm in a Synergy HT spectrophotometer (BIO-TEK, Winooski, Vermont, USA). Triplicate measurements were carried out with each sample.

### Determination of superoxide dismutase (SOD) activity

Brains were homogenized with cold lysis buffer (10 mM Tris, pH 7.5, 150 mM NaCl, 0.1 mM EDTA, 0.5% Triton-100). Superoxide dismutase (SOD; E.C: 1.15.1.1) activity was determined using a xanthine/xanthine oxidase (XOD) and NBT-based assay system which generates O_2_•^−^ that interacts with NBT to produce water-soluble formazan dye (OxiSelect SOD assay kit, Cell Biolabs). Sample absorbance was read at 490 nm in a Synergy HT spectrophotometer microplate reader (BIO-TEK, Winooski, Vermont, USA). All samples were measured at least three times.

### Determination of total glutathione and glutathione disulfide in brain

Frozen brain tissue samples were homogenized on ice using ultrasound in a 3-fold (w/v) volume of 1% (w/v) sulfosalicylic acid. Samples were centrifuged for 1 min at 12,000 g and 4°C. The supernatant was used for quantification of total glutahtione (GSx  =  amount of GSH plus twice the amount of GSSG) as described previously [65] in microtiter-plates according to the colorimetric Tietze method [66].

### Western Blotting

Nuclear extracts of brain cells were prepared according to Semenza and Wang (1992) [67] with some modifications after Soitamo et al. (2001) [68]. Brains were washed twice with cold PBS, and nuclear extracts were prepared with buffers containing 0.5 mM dithiothreitol, 0.4 mM phenylmethylsulfonyl fluoride (PMSF), 2 µg/ml of leupeptin, 2 µg/ml of pepstatin, 2 µg/ml of aprotinin, and 1 mM sodium vanadate. Isolated nuclear extracts were disrupted in 5 µl/ml Sigma protease inhibitor cocktail (Sigma, St. Louis, MO, USA) added homogenization buffer (10 mM Tris-Cl, 10 mM KCl, 1.5 mM MgCl_2_, 5 mM DTT, 5 µg/ml antipain, 1 mM Na_3_VO_4_, 1 mM dimethyloxallyl glycine (DMOG), 0.5 mM PMSF, 5 mM β-mercaptoethanol, pH 7.8) and centrifuged at 16000 g, 30 min, 4°C to remove debris. The protein content of the samples was determined according to Bradford (1976) [69] using bovine serum albumin as a standard. Supernatants (a volume equivalent to 20 µg protein) were subjected to 10% sodium dodecylsulfate-polyacrylamide gel electrophoresis. The separated proteins were subsequently transferred to polyvinylidene difluoride membranes (Millipore, Billerica, MA, USA). After blocking in 5% nonfat milk, the blots were incubated with a polyclonal antibodies directed against the C-terminus (PQEQVEQGKKLKAS) of eelpout HIF-1α (diluted 1∶1000) and a mouse anti-chicken α-tubulin (diluted 1∶2000) (Sigma, St Louis, MO, USA), respectively. Afterwards, the membranes were washed and incubated for 2 h at room temperature with horseradish peroxidase conjugated anti-rabbit or anti-mouse secondary antibody (Amersham Pharmacia Biotech, diluted 1∶150). After washing the membranes, the signals were detected by enhanced chemiluminescence (ECL, Amersham Pharmacia Biotech). The differences between each band intensity were compared using a commercial software package (Image-Pro Plus 7.0, Media Cybernetics, Silver Spring, MD, USA).

### Statistical analysis

Values are presented as the mean ± standard deviation (SD) and compared using Student's *t*-test or one-way analysis of variance (ANOVA) with Tukey's pairwise comparison. *Indicates a significant difference between treatment group and from the respective control one (*p*<0.05). Different and non-overlapping letters indicate significant differences between treatments (*p*<0.05), whereas overlapping letters indicate no significant differences between treatments (*p*>0.05).

## Supporting Information

Table S1 Primers used for qRT-PCR and *in situ* probe construction. F, forward primer; R, reverse primer.
(DOC)

Table S2 List of species and accession numbers for UCP sequences. * Sequences obtained form the Ensembl database (version 58).
(DOC)
