# Supplementary material for: Exploring Uncoupling Proteins and Antioxidant Mechanisms under Acute Cold Exposure in Brains of Fish
Source: PLoS One. 2011 Mar 25;6(3):e18180. doi: 10.1371/journal.pone.0018180 (PMC3064598; doi:10.1371/journal.pone.0018180)
Supplement: Table S1 — Primers used for qRT-PCR and in situ probe construction. F, forward primer; R, reverse primer. (DOC) [file pone.0018180.s001.doc]

**Supporting Information S1**

**Table S1.** Primers used for qRT-PCR and *in situ* probe construction

| **qRT-PCR**  **primer name** | **Primer sequence** | **Product size (bp)** |
| --- | --- | --- |
| QzUCP1 | F 5’-ACACAAACGGACTCAGACGA-3’  R 5’-TGCTACGCTGAACTCATCAA-3’ | 112 |
| QzUCP2 | F 5’-CTAAACCGGTTAGGGTGGAG-3’  R 5’-TTACAGTTGCAGCCGAAATC-3’ | 104 |
| QzUCP2l | F 5’-CGCCTTTCTGAACACACAACATC-3’  R 5’-TGACCTTTGGCCTTACAACACTG-3’ | 150 |
| QzUCP4 | F 5’-CAGCCTCGGGATTCAAAC-3’  R 5’-AAGACCAGGGAGCCATTCTA-3’ | 139 |
| QzUCP5 | F 5’-ACTCCTGGCAGCTACCGTAT-3’  R 5’-CTCAAGCCAGTGTGGAGAAA-3’ | 165 |
| QzCAT | F 5’-TAAAGGAGCAGGAGCGTTTGGCTA-3’  R 5’-TTCACTGCGAAACCACGAGGATCT-3’ | 177 |
| QzPPARαa | F 5’-ATTATGTACAGCCCTCTGAGCGGA-3’  R 5’-TGAGAACACTTCTGAGGACGGACT-3’ | 195 |
| QzPPARαb | F 5’-GTAGACATGGAGAATCGCTACCGT-3’  R 5’-AAGCTGCTGAGAGCGTCTTCAT-3’ | 134 |
| QzPPARδa | F 5’-AATGCCAGTTCTGCCGCTTTCAGA-3’  R 5’-TTCAGGTAGGCGTTGTTGACCCGTTT-3’ | 193 |
| QzPPARδb | F 5’-AATGCCAGTACTGCCGCTTTCAGA-3’  R 5’-TGTTGACGTGCTTGGCCAGTGTTT-3’ | 177 |
| QzPPARγ | F 5’-TCCACAGTTTGCAGAGAACAGCGT-3’  R 5’-TTGGCACTTGTTGCGGCTCTTCTT-3’ | 235 |
| QzGLUT1a | F 5’-CCATTTCTCCTGGGCTTTACCTTTA-3’  R 5’-CAGATTTGGCTTTGCTTTCCTCGTT-3’ | 121 |
| QzGLUT3 | F 5’-TCGTCAATGTCTTGGCTCTG-3’  R 5’-CAACATACATTGGCGTGAGG-3’ | 141 |
| QzRPL13A | F 5’-CCTCGGTCGTCTTTCCGCTATTG-3’  R 5’-CAGCCTGACCCCTCTTGGTTTTG-3’ | 248 |
| ***in situ* probe primer name** | **Primer sequence** | **Product size (bp)** |
| zUCP1_IS | F 5’-CAACTGCACAGAACTGGTGTCTTACGATCT-3’  R 5’-ATAGCCATACACCCAAAGCACTTCACAAT-3’ | 1201 |
| zUCP2_IS | F 5’-GCACGAGTTCTCAACGTTTCTCTATGGAAGA-3’  R 5’-TGGCCCGTTTCAGCTGCTCGTAAGT-3’ | 1110 |
| zUCP2l_IS | F 5’-GCAAGTGTTGTGCTGCTGTTATGCTGCTTT-3’  R 5’-GGGATTCCTCCATTCTCACAACAGAGCGTT-3’ | 1208 |
| zUCP4_IS | F 5’-TTCAGGCTCGTGGTTCCTTTGGGACATAGT-3’  R 5’-AAAGACCAGGGAGCCATTCTAAACCACGTTG-3’ | 976 |
| zUCP5_IS | F 5’-ACGAGAGGAAGGCGTCCGGGCATTGT-3’  R 5’-GGAGTCTGCCGACCTCAAATATTCCTCAGGG-3’ | 1200 |

F, forward primer; R, reverse primer
