# Supplementary material for: Exploring Uncoupling Proteins and Antioxidant Mechanisms under Acute Cold Exposure in Brains of Fish
Source: PLoS One. 2011 Mar 25;6(3):e18180. doi: 10.1371/journal.pone.0018180 (PMC3064598; doi:10.1371/journal.pone.0018180)
Supplement: Table S2 — List of species and accession numbers for UCP sequences. * Sequences obtained form the Ensembl database (version 58). (DOC) [file pone.0018180.s002.doc]

**Supporting Information S2**

**Table S2. List of species and accession numbers for UCP sequences**

| **Class** | **Species name** | **Protein name** | **Accession number** |
| --- | --- | --- | --- |
|  | *Ciona intestinalis* | Sea squirt UCPX | XP_002129757 |
| SLC25A7 | *Homo sapiens* | Human UCP1 | NP_068605 |
|  | *Mus musculus* | Mouse UCP1 | NP_033489 |
|  | *Ornithorhynchus anatinus* | Platypus UCP1 | XP_001512700 |
|  | *Xenopus tropicalis* | Frog UCP1 | NP_001107354 |
|  | *Cyprinus carpio* | Carp UCP1 | AAS10175 |
|  | *Fugu rubripes* | Pufferfish UCP1 | ENSTRUP00000033443* |
|  | *Danio rerio* | Zebrafish UCP1 | NP_955817 |
| SLC25A8 | *Homo sapiens* | Human UCP2 | NP_003346 |
|  | *Mus musculus* | Mouse UCP2 | NP_035801 |
|  | *Monodelphis domestica* | Opossum UCP2 | XP_001362966 |
|  | *Xenopus tropicalis* | Frog UCP2 | NP_989179 |
|  | *Oncorhynchus mykiss* | Trout UCP2A | ABC00183 |
|  | *Oncorhynchus mykiss* | Trout UCP2B | ABC00185 |
|  | *Cyprinus carpio* | Carp UCP2 | CAB46248 |
|  | *Tetraodon nigroviridis* | Tetraodon UCP2 | GSTENP00022194001* |
|  | *Tetraodon nigroviridis* | Tetraodon UCP2-like | GSTENT00022195001* |
|  | *Fugu rubripes* | Pufferfish UCP2 | ENSTRUP00000037074* |
|  | *Fugu rubripes* | Pufferfish UCP2-like | ENSTRUP00000037001* |
|  | *Danio rerio* | Zebrafish UCP2 | NP_571251 |
|  | *Danio rerio* | Zebrafish UCP2-like | NP_956647 |
| SLC25A9 | *Homo sapiens* | Human UCP3s  (short variant) | NP_073714 |
|  | *Homo sapiens* | Human UCP3l  (long variant) | NP_003347 |
|  | *Mus musculus* | Mouse UCP3 | NP_033490 |
|  | *Monodelphis domestica* | Opossum UCP3 | XP_001368096 |
|  | *Ornithorhynchus anatinus* | Platypus UCP3 | XP_001512822 |
|  | *Gallus gallus* | Chicken UCP3 | NP_989438 |
| SLC25A27 | *Homo sapiens* | Human UCP4 | NP_004268 |
|  | *Mus musculus* | Mouse UCP4 | NP_082987 |
|  | *Monodelphis domestica* | Opossum UCP4 | XP_001368742 |
|  | *Xenopus tropicalis* | Frog UCP4 | ENSXETP00000035487* |
|  | *Danio rerio* | Zebrafish UCP4 | NP_956635 |
|  | *Ciona intestinalis* | Sea squirt UCP4 | XP_002125994 |
| SLC25A14 | *Homo sapiens* | Human UCP5 | NP_003942 |
|  | *Mus musculus* | Mouse UCP5 | NP_035528 |
|  | *Monodelphis domestica* | Opossum UCP5 | XP_001381468 |
|  | *Gallus gallus* | ChickenUCP5 | NP_001012901 |
|  | *Fugu rubripes* | Pufferfish UCP5 | ENSTRUP00000023155* |
|  | *Danio rerio* | Zebrafish UCP5 | NP_956458 |

* Sequences obtained form the Ensembl database (version 58)
